# Selectivity Determinants of RHO GTPase Binding to IQGAPs

**DOI:** 10.3390/ijms222212596

**Published:** 2021-11-22

**Authors:** Niloufar Mosaddeghzadeh, Kazem Nouri, Oliver H. F. Krumbach, Ehsan Amin, Radovan Dvorsky, Mohammad R. Ahmadian

**Affiliations:** 1 Medical Faculty, Institute of Biochemistry and Molecular Biology II, University Hospital Düsseldorf, Heinrich Heine University Düsseldorf, 40225 Düsseldorf, Germany; mosaddeg@uni-duesseldorf.de (N.M.); Kazem.nouri@uhnresearch.ca (K.N.); o.krumbach@gmail.com (O.H.F.K.); ehsan.amin@hhu.de (E.A.); radovan.dvorsky@gmail.com (R.D.); 2 Department of Pathology and Molecular Medicine, McMaster University, Hamilton, ON L8S 4L8, Canada; 3 Medical Faculty, Institute of Neural and Sensory Physiology, University Hospital Düsseldorf, Heinrich Heine University, 40225 Düsseldorf, Germany

**Keywords:** IQGAP, scaffold, RHO GTPases, CDC42, RAC1, selective bindings

## Abstract

IQ motif-containing GTPase-activating proteins (IQGAPs) modulate a wide range of cellular processes by acting as scaffolds and driving protein components into distinct signaling networks. Their functional states have been proposed to be controlled by members of the RHO family of GTPases, among other regulators. In this study, we show that IQGAP1 and IQGAP2 can associate with CDC42 and RAC1-like proteins but not with RIF, RHOD, or RHO-like proteins, including RHOA. This seems to be based on the distribution of charged surface residues, which varies significantly among RHO GTPases despite their high sequence homology. Although effector proteins bind first to the highly flexible switch regions of RHO GTPases, additional contacts outside are required for effector activation. Sequence alignment and structural, mutational, and competitive biochemical analyses revealed that RHO GTPases possess paralog-specific residues outside the two highly conserved switch regions that essentially determine the selectivity of RHO GTPase binding to IQGAPs. Amino acid substitution of these specific residues in RHOA to the corresponding residues in RAC1 resulted in RHOA association with IQGAP1. Thus, electrostatics most likely plays a decisive role in these interactions.

## 1. Introduction

**Figure 1:**
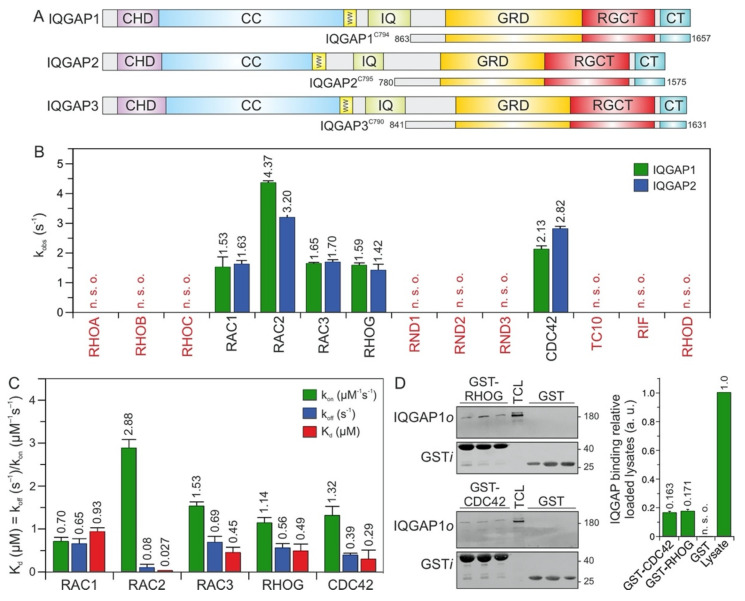
IQGAP1 and IQGAP2 selectively associate with CDC42 and RAC1-like proteins. (**A**) Domain organization of the IQGAP paralogs and their C-terminal fragments assessed in this study (see text for more details). (**B**) The association of IQGAP1^C794^ and IQGAP2^C795^ (2 µM) with various mGppNHp-bound RHO GTPases (0.2 µM) was investigated (Figure S1). The k_obs_ values for the interaction of IQGAP1 and IQGAP2 with several RHO GTPases, shown as bars, illustrate that both IQGAPs associate with CDC42 and RAC1-like proteins. The RHO-like proteins RND1, RND2, RND3, TC10, RIF, and RHOD did not associate with these IQGAPs under these conditions. (**C**) The association rates (k_on_) were measured using 0.2 µM mGppNHp-bound RHO GTPases with increasing concentrations (2–8 µM) of IQGAP1^C794^. Dissociation rates (k_off_) were measured by mixing 2 µM IQGAP1^C794^ complexed with mGppNHp-bound RHO GTPases (0.2 µM) and unlabeled RAC1-GppNHp (10 µM). The individual rate constants were calculated for the interaction of IQGAP1^C794^ with RAC- and CDC42-like proteins, and the results are plotted in bar charts. Association rates (k_on_), dissociation rates (k_off_), and dissociation constants (K_d_) for IQGAP1^C794^-RHO protein binding are shown. RAC2 showed the highest binding affinity for IQGAP1^C794^, followed by CDC42, RAC3, RHOG, and RAC1. The data are expressed as the means ± S.D. All measurements were obtained in duplicate. n. s. o. = no signal observed. Kinetic data, which are summarized in Table S1 and shown in Figure S2, were obtained in triplicate. The data are expressed as the means ± S.D. (**D**) Binding of endogenous IQGAP1 to GppNHp-bound RHOG and CDC42 (left panel) was analyzed in a GST pull-down assay (*n* = 3) using total cell lysate (TCL) of HEK-293 cell (i, input; o, output). GST-CDC42•GppNHp was used as positive control. GST control experiments confirmed the specificity of the interaction between RHOG and IQGAP1. The upper part of the membrane was used for an anti-IQGAP1 immunoblotting, and the lower for an anti-GST. Densitometry analysis of relative IQGAP binding to GST-CDC42 or GST-RHOG (a. u., arbitrary unit) were performed in the next step. Bar charts at the right panel display the quantitation of detected signal in GST-pull down assay from a triplicate experiment.

IQ motif-containing GTPase-activating proteins (IQGAPs) belong to the class of multidomain scaffold proteins that play central roles in the assembly of protein complexes and signaling networks [1,2,3,4,5,6,7]. In humans, three IQGAP paralogs have been described. The ubiquitously expressed IQGAP1 is the best-characterized paralog. IQGAP2 is mostly expressed in the liver, prostate, kidney, thyroid, stomach, testis, platelets, and salivary glands, while IQGAP3 is found in the brain, lung, testis, and intestine [8]. Multiple domains enable IQGAPs to interact with a large number of proteins and to modulate the spatiotemporal distributions of distinct signal-transducing protein complexes, including B/CRAF-MEK1/2-ERK1/2 [9,10,11], FGFR1-CDC42-NWASP-ARP2/3-actin [12,13,14], TIAM1-RAC1-PAK6 [15,16], and CDC42/RAC1/CLIP170 [17,18]. IQGAP paralogs share similar domain organization and high sequence homology (Figure 1A). The N-terminal calponin homology domain (CHD) binds F-actin [19]. The polyproline-binding region (WW) binds ERK1/2 [9]. The IQ motif (IQ) binds HER1/2, KRAS, B/CRAF, MEK1/2, and calmodulin [4,20,21,22,23,24]. The RASGAP-related domain (GRD) and RASGAP C-terminal domain (RGCT) bind to CDC42 and RAC1. The C-terminal domain (CT) binds E-cadherin, β-catenin, APC, and CLIP170 [3].

CDC42 and RAC1 belong to the RHO GTPase family, which includes 20 classical paralogs [25] that control diverse cellular functions [26,27]. RHO GTPases are classified into six subfamilies: the RHO subfamily (RHOA, RHOB, and RHOC); the RAC subfamily (RAC1, RAC1B, RAC2, RAC3, and RHOG); the CDC42 subfamily (CDC42, G25K, TC10, TCL, WRCH1, and WRCH2); the RND subfamily (RND1, RND2, and RND3); and RHOD, RIF, and RHOH, which do not precisely fall into any of these subfamilies [25,28]. The RHOBTB and MIRO subfamilies are atypical members of the RHO family that are structurally different from classical RHO family members and possess other additional functional domains [29].

RHO GTPases are molecular switches that cycle between an inactive (GDP-bound) and an active (GTP-bound) form [28]. In the active state, they interact with a multitude of target (effector) proteins, such as IQGAPs, to induce cellular responses [30,31,32]. Interaction with RHO GTPases, such as CDC42 or RAC1, and/or phosphorylation of Ser-1441 and Ser-1443 may release IQGAPs from an autoinhibited state and induce their activated signaling competent state [20,22,23,33]. The interaction of the C-terminal half of IQGAP1, encompassing the GRD, RGCT, and CT domains (hereafter called C794), with RAC1 and CDC42 has been intensively studied by several groups [21,23,33,34,35,36,37,38,39]. Despite the common binding properties of CDC42 and RAC1 to IQGAPs, there are significant differences, which may be attributed to divergent IQGAP-RHO GTPase complexes that control distinct cellular processes [23,24,36,40,41]. As the highly flexible switch I and II regions (encompassing amino acids 29–42 and 62–68, respectively), which change their conformation upon GDP to GTP exchange [31], are almost identical in CDC42 and RAC1, the selectivity-determining residues need to be located outside these two regions.

Since its discovery in 1994, IQGAP1 has emerged as a key scaffold protein [42] that links crucial components of multiple cellular processes. Many studies have provided valuable evidence for the interaction between IQGAP1 and CDC42, but the mechanism determining IQGAP binding selectivity for different members of the RHO GTPase family has remained unclear. The following questions were addressed in this study: To what extent do IQGAP paralogs differ in their RHO GTPase-binding characteristics and specificity, and how does IQGAP1 distinguish different RHO GTPases? To this end, we investigated the interactions of IQGAPs with 14 RHO GTPases using the C794 and C795 segments of IQGAP1 and IQGAP2, respectively, which exhibit a sequence identity of 72%. We excluded IQGAP3, RHOH, WRCH1, WRCH2, and TCL from this study because of their low solubility and physical instability. These analyses revealed that IQGAPs bind CDC42 and RAC1-like proteins but not RHO-like proteins. IQGAP1 competition experiments along with mutational and structural analyses revealed three distinct regions proximal to the switch regions that are differentially involved in selective binding of IQGAP1 and 2 to RHO GTPases.

## 2. Results

### 2.1. IQGAP1/2 Selectively Bind CDC42 and RAC1-Like Members of the RHO Family

The C-terminal 794 amino acids (aa) of IQGAP1, encompassing the GRD, RGCT, and CT domains, and C795 of IQGAP2, were successfully purified to measure their binding properties over a broad range of RHO GTPases. Interaction studies were performed using time-resolved stopped-flow fluorescence (SFF) spectrometry under previously described conditions [23]. Accordingly, both IQGAPs were associated similarly with the active forms of RAC1, RAC2, RAC3, RHOG, and CDC42, but not with RND1, RND2, RND3, TC10, RHOA, RHOB, RHOC, RHOD, or RIF (Figure 1B and Figure S1A–F).

RND proteins represent a distinct group of proteins within the RHO family. They were purified in their GTP-bound state, but replacing GTP with mGppNHp, 2′/3′-O-(N-methyl-anthraniloyl)- guanosine-5′-[(β,γ)-imido]triphosphate, a slow-hydrolyzing analog of GTP, resulted in complex instability. Therefore, for interaction studies with IQGAPs, we performed indirect competitive assays. We measured the association of IQGAP1^C794^ with RAC1 in the presence and absence of excess GTP-bound RND proteins. As a positive control, we used GppNHp-bound CDC42. In contrast to CDC42, which competitively blocked the IQGAP-RAC1 interaction, no binding of RND proteins was observed (Figure 1B and Figure S1G,H), suggesting that IQGAP1 and IQGAP2 do not interact with these unconventional members of the RHO family.

Given these findings, it was important to investigate the complex formation and binding stoichiometry between CDC42/RAC1 and the IQGAP proteins. LeCour et al. have proposed that constitutively active CDC42^Q61L^ but not RAC1^Q61L^ binds the IQGAP2 (GAP)-related domain (GRD) in a 2:1 ratio to promote IQGAP2 dimerization [41]. Therefore, we performed analytical size-exclusion chromatography using IQGAP1^C794^ and IQGAP2^C795^ alone or mixed with CDC42•GppNHp or RAC1•GppNHp. The elution profiles showed that CDC42, RAC1, and IQGAP1 eluted as dimers, while IQGAP2 eluted mainly as monomers and to some extent as trimers and tetramers (Figure S3A; peaks #1 and #2). The elution profiles of the IQGAPs mixed with CDC42 and RAC1 showed, in addition to RAC1 and CDC42 (peak #1), two peaks (#3 and #5), indicating molecular weights (*M*_W_s) of 222–235 kDa and elution volumes of 10.2–11.0 mL (Figure S3A). Coomassie-brilliant-blue-stained SDS-PAGE gels revealed that only peak #3 contained IQGAP complexes with RAC1 and CDC42, with an average *M*_W_ of 228 kDa that corresponds to a heterotetramer (Figure S3B). IQGAP2^C795^ also eluted as higher oligomers (peak #6), which did not contain either RAC1 or CDC42.

LeCour et al. have reported a high affinity interaction between CDC42^Q61L^ and IQGAP GRD (41). In our previous study, we have shown that CDC42^Q61L^ has a 13-fold stronger interaction with GRD as compared with CDC42^WT^ (23). Therefore, we purified and investigated the stoichiometry of CDC42^Q61L^•GppNHp for its complex formation with IQGAP1 GRD in direct comparison with CDC42^WT^•GppNHp. In the case of CDC42^Q61L^, the elution profile represented two peaks (Figure S4, upper middle panel) for the GRD and CDC42^Q61L^ complex, corresponding to heterotrimeric complex with a stoichiometry of 2:1, as proposed by LeCour et al. [23,41]. However, GRD and CDC42^WT^ complex eluted as a heterotetramer (a 2:2 complex; Figure S4, lower panels).

Overall, the analyses of the size-exclusion chromatography data suggest that under our experimental conditions, the composition of the IQGAP1/2 complexes with both RAC1 and CDC42 corresponds to a 2:2 ratio. Furthermore, the CDC42-GppNHp elution profile at 15.6 and 15.9 mL of elution volume (Figure S3) confirmed the previous observations reported by Zhang et al. regarding the reversible homodimerization of RHO family GTPases [43].

### 2.2. RAC2 Exhibited the Highest Affinity for IQGAP1

To examine binding properties, the respective association rate constants (k_on_) and the dissociation rate constants (k_off_) were determined for the interaction of IQGAP1^C794^ with CDC42 and RAC1-like proteins under the aforementioned conditions (Figure 1C and Figure S2). All the kinetic parameters along with calculated dissociation constants (K_d_) are summarized in Table S1. The values are in a range similar to that of wild-type RAC1, RAC3, RHOG, and CDC42, with the exception of RAC2, which strikingly showed a K_d_ value of 27 nM, the highest affinity for IQGAP1^C794^ (Figure 1C). The rapid association and slow dissociation rates are remarkable, and suggest that the RAC2–IQGAP1 interaction remains stable for a long residence time.

The IQGAP1^C794^ binding of RHOG, in addition to its binding to the RAC and CDC42 proteins, prompted us to investigate the association of RHOG with endogenous IQGAP1 using purified GST-RHOG•GppNHp as bait in a pull-down assay. GST was used as the negative control, and GST-CDC42•GppNHp was used as the positive control. Quantification of the immunoblot analysis using specific antibodies against GST and IQGAP1 showed that cellular IQGAP1 bound RHOG as efficiently as it bound CDC42 (Figure 1D).

Next, we performed an in-depth investigation of the IQGAP1^C794^ interactions with RAC1 and CDC42, which are widely acknowledged to be IQGAP-binding partners.

### 2.3. Potential Hotspots for IQGAP Binding Appear Outside the Switch Regions

**Figure 2:**
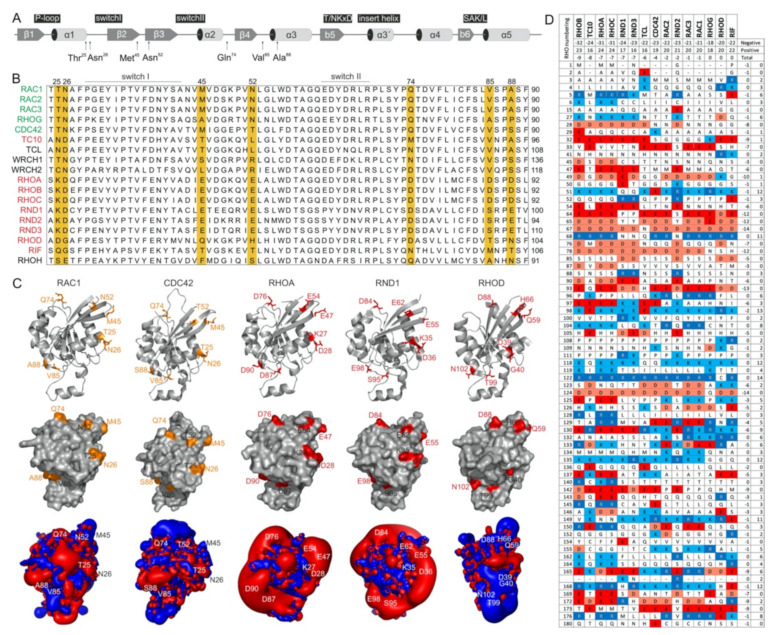
RHO GTPases exhibit significantly different electrostatic properties. (**A**) The G domain organization of RAC1 indicates secondary structure elements, key functional regions and locations of residues crucial for IQGAP1 binding. (**B**) A multiple amino acid sequence alignment of canonical RHO GTPases revealed various residues outside of the switch regions that may determine their differential interactions with IQGAPs. IQGAP binders are colored green, and the nonbinders are colored red. (**C**) Structures in ribbon representation, solvent accessible proteins surfaces and electrostatic potential maps for RAC1 (PDB code, 1MH1), CDC42 (PDB code, 2QRZ), RHOA (PDB code, 1A2B), RND1 (PDB code, 2CLS), and RHOD (PDB code, 2J1L) are shown. Thr-25, Asn-26, Met-45, Asn-52, Gln-74, Val-85, and Ala-88 of RAC1 proposed to determine its specificity for the binding of IQGAPs are located on the surface, negatively charged residues on corresponding positions in, for example, RHOA and RND1 cause significant negative electrostatic potentials. Images were generated with the PyMOL molecular viewer. (**D**) The distribution of charged amino acids vary significantly among RHO GTPases despite their high sequence homology. Sequence alignment of the RHO GTPases used in this study reduced in a way that only loci containing at least one positively charged amino acid, i.e., arginine or lysine, or one negatively charged amino acid, i.e., glutamate or aspartate, were retain, respectively. It demonstrates diverse occurrence of charges in proteins molecules of RHO GTPases that is also reflected on huge differences of electrostatic potentials shown in C. They roughly also correspond to theoretical net charges for whole proteins that were obtained as sums of the +1 or −1 for positively or negatively charged residues, respectively. As only RHOoD and RIF were found to be electrically neutral while all other GTPases possess overall negative net charge, characteristic lobes of negative, red colored electrostatic potentials around the majority of proteins were observed (for reference see also Figure S5).

The switch regions (Figure 2A), which are generally known as effector binding sites, are required but not sufficient for effector binding selectivity. The amino acid sequences of these two regions are almost identical, which is particularly notable in comparison to IQGAP1-binding proteins (e.g., members of the RAC subfamily) with nonbinders (e.g., members of the RHO subfamily) (Figure 2B). Thus, a set of specificity-determining residues in RHO GTPase that direct interactions with IQGAPs must reside outside of the switch regions. In this context, notably, the CDC42 subfamily includes both IQGAP binders and nonbinders. Accordingly, we found four different hotspots, residues 25/26, 45/52, 74, and 85/88 (based on CDC42/RAC numbering), that are highly conserved in IQGAP1 binders and clearly deviate from the corresponding residues in nonbinders (Figure 2B). Notably, we did not consider residues that are quite variable not only between the binders and nonbinders but also within the IQGAP1 binders themselves (e.g., T24, A27, G30, S41; Figure 2B). Moreover, an inspection of the crystal structures of RHO GTPases in five different subfamilies showed that these four sites did not significantly contribute to local structural variations (Figure 2C, upper panel). These residues surround the switch regions and, most interestingly, are all located on the surface of the respective proteins and are thus available for intermolecular interactions (Figure 2C, middle panel).

As almost all amino acids at the selected hotspots in RHOA and RND proteins have charged side chains, electrostatics very likely play a crucial role in complex formation with IQGAP1. With the aim of verifying this hypothesis, we first calculated the electrostatic potentials around these molecules. While the form and magnitude of the electrostatic isosurfaces for cognate RHO GTPases were found to be similar, striking differences were found between their subclasses, with particularly strong negative potentials in the cases of TC10, TCL, RHOA, and RND proteins (Figure 2C, lower panel). The electrostatic surface potentials of 15 different RHO GTPases are shown in Figure S5. Aiming to understand the origin of these differences in the electrostatic potentials of the 15 examined RHO GTPases, we calculated the net charges of their G-domains with −1 attributed to aspartic or glutamic acid and +1 attributed to arginine or lysine. Although RHO GTPases are highly homologous, variations in particular amino acids that might seem negligible from a sequence point of view can lead to a broad span of net protein charges. In the cases of the studied GTPases, the span of electrostatic charges ranges from −9 for RHOB to electrically neutral RIF, clearly explaining the differences in electrostatic potential. The larger the lobe of the negative potential around the protein is, the more negative its net charge. Correlating the electrostatic charge with the binding to IQGAP1, negative charges might discriminate the association with TC10, TCL, RHOA, and RND paralogs. On the other hand, balanced potentials seem to be just a prerequisite for binding, because the charges and corresponding electrostatic potentials of all other GTPases are similar, but RHOD and RIF belong to nonbinders.

### 2.4. PAK1, p50^GAP^, and DOCK2 Compete with IQGAP1 for Binding RAC1

**Figure 3:**
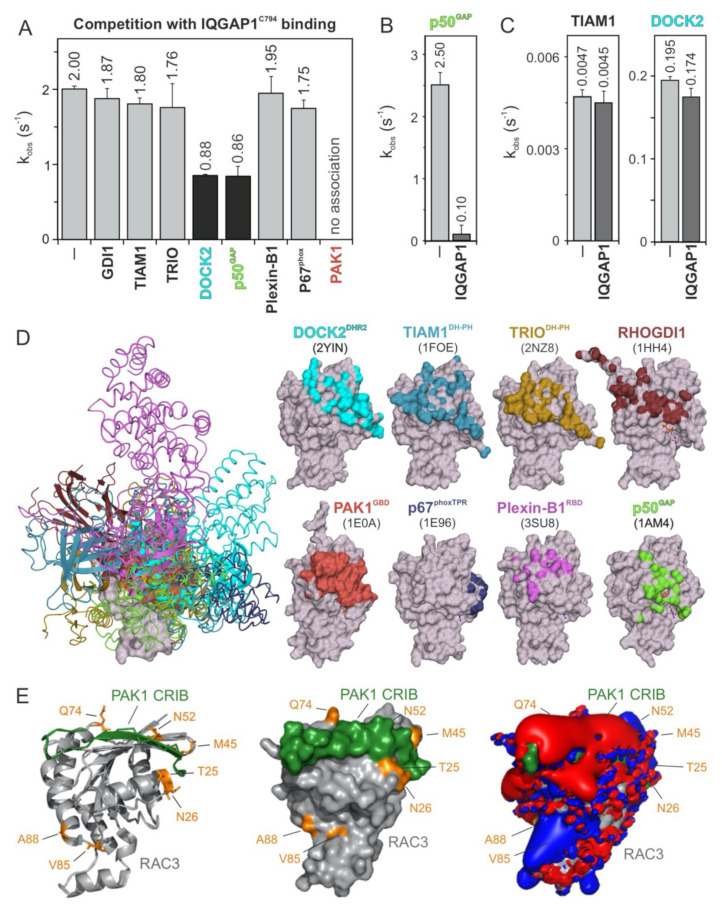
IQGAP1^C794^ competes with DOCK2, p50^GAP^, and PAK1 for binding RAC1. (**A**) The evaluated observed rate constants (k_obs_), shown as bars, demonstrate that IQGAP1^C794^ associates with RAC1 regardless of the presence of excess amounts of GDI1, TIAM1, TRIO, Plexin-B1, or p67^phox^, while the association was blocked in the presence of DOCK2 or p50^GAP^ and completely abolished in the presence of PAK1. (**B**) The p50^GAP^-stimulated GTPase activity of RAC1 was drastically reduced in the presence of IQGAP1^C794^. (**C**) TIAM1- and DOCK2-catalyzed nucleotide exchange activity of RAC1 was not significantly changed in the presence of excess amounts of IQGAP1^C794^. All measurements in (**A**–**C**), which are shown in detail in Figure S6, were obtained in triplicate. The data are expressed as the means ± S.D. (**D**) The left panel shows the structure of RAC1 (gray represents the surface) in complex with different RAC and CDC42 interacting partners (in different colored ribbons), including DOCK2^DHR2^, TIAM1^DH-PH^, TRIO^DH-PH^, p50^GAP^, GDI1, PAK1^GBD^, p67^phoxTRP^, and Plexin-B1^RBD^. The right panel highlights the contact sites of these binding proteins on the surface of RAC1 in the corresponding colors. The protein database identification codes of the respective structures are indicated. (**E**) The complex structure of RAC3 (PDB code, 2IC5) and the CRIB motif of PAK1 (PDB code, 2QME) shows that T25, N26, M45, N52, and Q74 of RAC3 are in close proximity to the CRIB motif-binding region. Electrostatic potentials (right panel) show that the PAK1 CRIB motif generates an overall negative electrostatic surface potential.

To further map the IQGAP1^C794^-binding regions on the surface of the RAC1 structure, we performed competitive binding experiments. We repeated the measurement of the IQGAP1^C794^ association with RAC1•mGppNHp in the absence and presence of a 10-fold molar excess of other RAC1-interacting proteins that may be competitors: full-length GDI1, the DBL homology-pleckstrin homology tandem (DH-PH) domain of TIAM1 and TRIO, the DOCK homology region 2 (DHR2) domain of DOCK2, the GAP domain of p50^GAP^, the GTPase-binding domain (GBD) of PAK1, the RAC1-binding domain of plexin-B1 RBD, and the tetratricopeptide repeat (TPR) of p67^Phox^ (Figure 3). These proteins were premixed with IQGAP1^C794^ before rapid mixing with RAC1•mGppNHp in a stopped-flow apparatus. The working model was based on the presumption that if the binding of RAC1 to IQGAP1^C794^ and to RAC1-interacting proteins is mutually exclusive, then the proteins in the mixture will interfere with the ability of IQGAP1^C794^ to associate with RAC1. As shown in Figure 3A and Figure S6, IQGAP1^C794^ association with RAC1•GppNHp was partially abolished with DOCK2 and p50^GAP^, completely abolished with PAK1, and not affected by the other proteins. Notably, GEFs, and most likely DOCK2, do not significantly distinguish between GDP- and GTP- (or GppNHp-) bound RHO GTPases [44].

In addition, we measured the impact of IQGAP1^C794^ binding to RAC1 on the GEF and GAP activities of TIAM1, DOCK2, and p50^GAP^ (Figure 3B,C). The speculation that GEFs may compete with IQGAP1^C794^ for RAC1•GDP binding is based on the assumption that IQGAP1^C794^ binds to other sites outside the switch regions [40]. No change was observed for the nucleotide exchange reaction catalyzed by TIAM1 or DOCK2 (Figure 3C), corroborating our previous observation that IQGAP1^C794^, which binds CDC42•GDP, does interact with RAC1•GDP [23]. In contrast, p50^GAP^-stimulated GTP hydrolysis activity was drastically inhibited, reduced by 25-fold, in the presence of IQGAP1^C794^ (Figure 3B), confirming the selective and high-affinity binding of IQGAP1^C794^ to RAC1•GTP.

To determine which amino acids of RAC1 are critical for the observed interactions and effects, we first overlaid the extracted structures of the investigated binding proteins (Figure 3D, left panel) with residues that form the interacting interfaces and depicted them on a surface representation of the respective RAC1 structures (Figure 3D, right panel). The interacting interfaces are shown in colors corresponding to the RAC1-binding proteins. We further analyzed the crystal structure of RAC3 in complex with PAK1 GBD, which fully interfered with IQGAP1^C794^ binding to RAC1, and may thus share overlapping binding regions. Remarkably, the residues previously identified by sequence structural analysis as potential (hot)spots for the association of RHO GTPases with IQGAP1^C794^, namely, T25, N26, M45, N52, and Q74 of RAC3, are located in proximity of the RAC1-binding region of PAK1 GBD (Figure 3E). Visualizing the electrostatic potential of this complex structure showed that PAK1 GBD generates an overall negative electrostatic potential on the surface of RAC3 (Figure 3E, right panel).

### 2.5. IQGAP1 Binding Hotspots Significantly Vary among RHO GTPases

**Figure 4:**
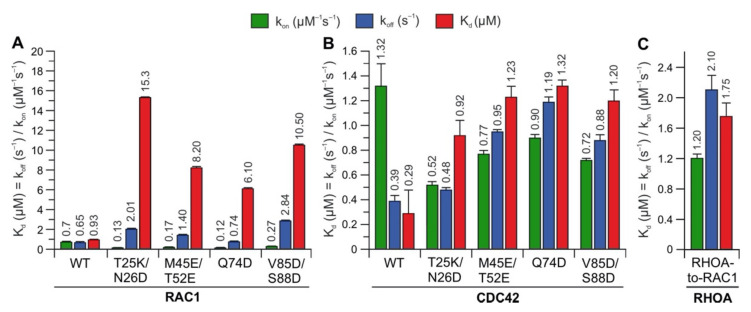
Kinetic measurements of RAC1 and CDC42 variants binding IQGAP1^C794^. The calculated association rates (k_on_), dissociation rates (k_off_), and dissociation constants (K_d_) for the interaction of IQGAP1^C794^ with different variants of RAC1 (**A**), CDC42 (**B**), and RHOA (**C**) are plotted as bar charts. All kinetic data are summarized in Table S1 and shown in Figure S7. The data are expressed as the means ± S.D.

To identify whether the predicted hotspots determine differences in the interaction of IQGAP1^C794^ with RHO proteins, we replaced these sites in RAC1 and CDC42 with the corresponding amino acids in RHOA (T25K/N26D, M45E/N52E, Q74D, and V85D/S88D) (Figure 2A). Notably, S88 of CDC42 is in the same region as IQGAP2-contacting residues [41]. The interaction of these variants with IQGAP1^C794^ was measured under the same conditions as described above. Strikingly, major changes in the IQGAP1^C794^ binding kinetics were observed for the RAC1 variants but not for the CDC42 variants (Figure 4, left and middle panels). All the variants exhibited slower association kinetics and faster dissociation kinetics (Figure S7). As a result, the overall decrease in the binding affinities of the RAC1 variants for IQGAP1^C794^ ranged between 7- and 17-fold, suggesting that these residues are either part of the RAC1–IQGAP1 binding interface or in close proximity to the IQGAP1-binding sites. To identify the impact of these residues, we generated a RHOA variant containing five substitutions, K27T, D28N, E47M, E54N, and D76Q, to mimic RAC1. Interestingly, this RHOA-to-RAC1 variant was capable of associating with IQGAP1^C794^, while RHOA^WT^ did not show any association with IQGAP1^C794^ (Figure 4, right panel). These data confirmed the identified sequence-specific binding sites as hotspots.

## 3. Discussion

A large number of studies have examined the interaction between IQGAPs and the small GTPases of the RHO family. Among the 20 classical RHO proteins, RAC1 and CDC42 have been extensively studied to characterize their binding behavior with IQGAPs [21,23,33,36,39,45]. Accordingly, IQGAPs are able to interact with different RHO GTPases. To understand the roles of these interactions in the orchestration of signaling events in which IQGAPs serve as scaffold proteins, we explored the selectivity of these interactions. Specifically, we measured the protein–protein interaction of 14 RHO GTPases with IQGAP1^C794^ and IQGAP2^C795^ to identify selectivity determinants. Time-resolved SFF spectrometry was performed to monitor the kinetics of IQGAP associations with RHO GTPases. The results clearly showed that these two IQGAP paralogs bind only CDC42, RHOG and RAC-like proteins. Notably, as RHOG belongs to the same branch of the phylogenetic tree of RHO GTPases that includes RAC1, RAC1B, RAC2 and RAC3, we suggest designating it RAC4.

The comparative analysis of the binding kinetics of the RAC paralogs RAC1, RAC2, and RAC3 with IQGAP1^C794^ showed that RAC2 kinetics with IQGAP1^C794^ were clearly different than those of RAC1 and RAC3, which is consistent with our previous studies. Notably, RAC1 and RAC3 have closely related biochemical properties that differ from those of RAC2. Previous studies reporting results of modeling and normal mode analyses supported the idea that the altered molecular dynamics of RAC2, particularly at the switch I region, may be critical for differences in its behaviors compared to those of RAC1 and RAC3 [46]. In our study, RAC2 exhibited a 4-fold faster k_on_ and an 8-fold lower k_off_, which resulted in a 34-fold lower K_d_ value, compared to RAC1 (Figure 1C). An amino acid sequence comparison of the RAC proteins showed that three identical residues in RAC1 and RAC3 were different in RAC2: S48, Y90, and D150 in RAC2 corresponded to G48, F90, and G150 in RAC1 and RAC3, respectively. These residues are located outside the IQGAP1 GRD-binding interface of two CDC42 molecules, which mainly contacts the switch regions [41]. This previous finding and the fact that CDC42 and RAC1 were found to differ in their interactions with IQGAP1 [23,24,40] suggest that RAC proteins may have additional contact sites that differ from those of CDC42. Casteel et al. and Bhattacharya et al. have shown the formation of an IQGAP1 complex with RHOA and RHOC but not with RHOB [47,48]. Our results do not confirm the direct binding of IQGAP1/2 to RHOA/C. In our opinion, the observed interactions of IQGAP1 with RHOA or RHOC appear to be indirect, since these proteins were coimmunoprecipitated from cells overexpressing tagged RHO wild-type proteins and their constitutively active form, or they may have been mediated by an IQGAP1 domain that does not include C794. Evidence supporting an indirect effect is based on our residue-swapping experiment: a variant of RHOA with five substitutions mimicking RAC1 was able to efficiently bind IQGAP1. In contrast, RHOA^WT^ did not bind IQGAP1, validating the identified sequence-specific binding sites as binding hotspots. Our findings also rule out the possibility that some domains other than C794 may mediate binding with RHOA.

Several proteins, including IQGAP3 and the RHO family members RHOH, WRCH1, WRCH2, and TCL, were not investigated in this study because of their limited solubility and stability. Our efforts to purify and characterize these proteins by generating various constructs, particularly IQGAP3, were not successful. In addition, the preparation of mGppNHp-bound RND proteins was not possible due to their instability in the presence of other guanine nucleotides. Purified RND proteins were exclusively GTP-bound, and attempts to hydrolyze GTP to GDP for several days at 25 °C or to exchange GTP for GTP analogs resulted in their precipitation [28,49]. The reason for this outcome is that these proteins are not regulated by a conventional GDP/GTP cycling mechanism and exist in the GTP-bound form in cells [28,49].

A remarkable feature of RHO GTPases is that their regulators (GDIs, GEFs, and GAPs) and effectors, although functionally quite diverse, share a consensus binding site encompassing switch I and II regions [31]. The competition experiments in our study were based on this basic concept (Figure 3A–C). In contrast to regulators that interact with RHO GTPases to modulate their switch function, the interaction between RHO GTPases and their effectors controls a wide range of intracellular signaling pathways and depends on the kinetics of their interactions, not their binding affinity. In fact, p67^phox^ and Plexin-B1, which bind RAC1 with slightly lower binding affinities of 2.7 and 6.6 µM, respectively [50,51], compared to IQGAP1^C794^, were unable to compete with IQGAP1^C794^, even at 10-fold molar excess. These proteins have an affinity for RAC1 similar to that of IQGAP1^C794^ but different kinetic properties upon binding (a fast k_on_ and a slow k_off_), as we determined in this study. The observed competitive effects of DOCK2 and p50^GAP^ on IQGAP1^C794^ binding to RAC1•GppNHp seem to be consistent with the presumed importance of the binding kinetics. A previous study showed that very fast GEF- and GAP-catalyzed reactions were both preceded by a much faster association with their cognate GTPase, implying very high k_on_ values [52].

Moreover, the structural properties that characterize the GTPase-binding domains of the effectors and their binding sites are, despite their fundamentally conserved sites, rather diverse. This includes the unexpected features of p67^phox^ contact sites on RAC1. p67^phox^ has an α-helical domain with four tetratricopeptide repeat (TPR) motifs [53] that bind α1, the N-terminal residues of switch I, and the G3 and G5 loops, but not the switch II region or the principal parts of switch I (Figure 3D) [51]. It has also been proposed that the switch regions might be the contact sites for a third protein that is simultaneously associated with the RAC1•GTP•p67^phox^ complex and bound to membrane phospholipids [54,55].

In a comprehensive study, Owen et al. analyzed a multitude of CDC42 and RAC1 variants, particularly variants with changes in the switch regions and the insert helix, to assess their interactions with IQGAP1. Their results suggested that CDC42 and RAC1 associate with IQGAP1 in a significantly different manner [36,40]. Major IQGAP-binding sites in CDC42 are residues in the switch regions, including P34, V36, F37, D38, D63, Y64, R66, and L67, which are basically identical in CDC42 and RAC1. This and another study have shown that the insert helix of CDC42, especially residue Asn-132, may provide an additional binding site for IQGAP2 GRD on CDC42, leading to CDC42 dimerization, which is not evident for RAC1 [24,41]. These data may explain the slightly different binding kinetics of CDC42 and RAC1 towards IQGAP1 (Figure 1B); the faster association rate and slow dissociation rate explain the 3-fold higher binding affinity of CDC42 for IQGAP1 compared to that of RAC1. Using analytical size-exclusion chromatography, we did not observe differences between RAC1 and CDC42 in their complex formation with IQGAP1 or IQGAP2 (Figure S3). All four protein complexes showed an average *M*_W_ of 228 kDa, which corresponds to heterotetramers formed in a 2:2 ratio. Our data on residue swapping in RHO GTPases successfully validated the role of hotspot residues identified in the RAC1–IQGAP1^C794^ interaction (Figure 4). Four of seven the hotspots (T25, T52, V85, and S88) investigated in the residue swapping experiments in this study are within the CDC42–IQGAP2 binding interface, while N26, M45, and Q74 are clearly outside but close to the IQGAP2-binding site [24,41]. Our findings support previous research on overlapping RAC1 and CDC42 contact regions, but they also provide additional insights into the possible RAC1–IQGAP interacting interface, which needs to be confirmed by additional structural studies.

LeCour et al. have investigated the IQGAP1 GRD domain in a complex with CDC42Q61L [23,41]. Different groups used this variant of RAC1, CDC42, and RHOA in biophysical studies because it binds to effector proteins with 10- to 30-fold higher affinity. The reason is a tremendous increase and stabilization of an exposed hydrophobic cluster between the switch region in GTP-bound proteins, consisting of P36, V38, and F39 from switch I; Q63, Y66, R68 from switch II; and L69 and L72 from helix α2 [31,56]. This is consistent with the determined binding affinity of IQGAP1 for CDC42Q61L•mGppNHp of 2.37 µM, which is 13-fold higher as compared to the K_d_ value obtained from IQGAP1 binding to CDC42 [23,41]. In conclusion, we believe that using this variant to characterize RHO GTPase–effector interaction may lead to incorrect conclusions.

In silico analyses of RHO GTPases in this study, based on sequence alignment, comparison of different crystal structures, and electrostatic potentials together with kinetic experiments revealed that significant differences in the IQGAP-binding selectivity can be attributed to a few amino acids deviating between subgroups in the RHO GTPase family. A critical issue that needs to be further considered involves the electrostatics that can either affect bimolecular interactions due to repulsive forces or substantially enhance molecular interactions based on attractive forces, which can contribute to the selectivity and rapid association of two molecules [57,58,59,60,61,62,63]. A structural inspection of RHO GTPases revealed that they differ considerably in their electrostatic potentials, as demonstrated by the equipotential contours; even highly related paralogs, such as CDC42 (with a net charge of −4) vs. TC10 (−8), RND1 (−7) vs. RND2 (−2), and RAC1 (−1) vs. RAC2 (−1), are quite different regarding their electrostatic potential distribution (Figure 2D and Figure S5). These differences may have significant effects on interaction selectivity. A remarkable example is the dramatic difference of the k_on_ values of approximately 800-fold for the WASP association with CDC42 and TC10. Unique glutamates in CDC42 (E49, E171, and E178), which are missing in TC10, generate favorable electrostatic steering forces that control the accelerated CDC42–WASP association reaction [45,58]. Notably, RHOC and RHOD are unrelated members of the RHO family with respect to their intrinsic nucleotide exchange and hydrolysis reactions [28] and their interactions with GEFs and GAPs [64,65]. Although RND1 and RND3 show surface potentials similar to RHOA (Figure S5), they are known to antagonize RHOA function [66,67]. A previously performed structure–function analysis showed that GTP-bound RND proteins share a similar fold but striking differences from the conventional members of the RHO family, such as RHOA, especially with regard to interacting interfaces with RHOA regulators and effectors [28,49]. Although a large computational toolbox is currently available for studying the roles played by electrostatics in the regulation of the protein life cycle and protein interactions, electrostatic features are still neglected factors in basic science [59].

Considering the high sequence identity between the RHO GTPases within the switch regions, which are generally acknowledged to be the main binding sites for three classes of regulators and of downstream effectors [31], we proposed that further contact sites outside of the switch regions are required to define the selectivity of the respective interactions. Recently, nine different CDC42 missense mutations causing a phenotype resembling Noonan syndrome have been identified by researchers. Among these mutants, *CDC42^R66G^* and *CDC42^R68Q^* exhibited defective interactions with IQGAP1 [45]. However, the mutated residues are part of the switch II region and do not significantly differ between RHO GTPases. Another recently described disease, called NOCARH syndrome, caused by a specific missense mutation in *CDC42*^R186C^, was identified by Lam and colleagues. Biochemical analysis has shown that the interaction of this mutant with IQGAP1 is dramatically diminished [39]. It was thus proposed that this mutant localizes to the Golgi apparatus, since IQGAP1 has been shown to promote CDC42 translocation from the Golgi apparatus to the plasma membrane [68].

In conclusion, our study demonstrated that, in addition to those in the switch regions, distinct residues in CDC42 and RAC1-like proteins are required for their association with IQGAP1^C794^, and these residues are missing in nonbinders. Since IQGAPs are involved in many cellular processes, it will be a great advantage to elucidate the respective mechanisms of their scaffolding functions. Our data shed light on the mechanism of RHO GTPase binding to IQGAPs, allowing us to better understand their physical interactions. The IQGAP1^C794^ interaction with CDC42 and RAC1 was found to be slightly different. These interactions remain a subject of further structural analysis. The binding characteristics of other RHO GTPases, including RAC2 and RHOG, to IQGAP proteins in macrophages and endothelial cells and their roles in differentiation, angiogenesis, barrier function, and inflammation await further investigation [69,70].

## 4. Materials and Methods

**Constructs.** Different variants’ pGEX vectors (pGEX2T and pGEX4T-1) encoding an N-terminal glutathione S-transferase (GST) fusion protein were used to overexpress human IQGAP1^C794^ (acc. no. P46940; aa 863–1657), IQGAP1^GRD^ (aa 962–1345), human Plexin B1 RBD (acc. no. O43157; aa 1724–1903), human p67^phox^ TRP (acc. no. P19878; aa 1–203), human PAK1 GBD (acc. No. Q13153; aa 57–141), murine TIAM1 DH-PH (acc. no. Q60610; aa 1033–1404), human TRION DH-PH (acc. no. O75962; aa 1226–1535), and human p50^GAP^ (acc. no. Q07960; aa 198–439), as well as human RHO-related genes, that is, RAC1 (acc. no. P63000; aa 1–179), RAC2 (acc. no. P15153; aa 1–192), RAC3 (acc. no. P60763; aa 1–192), RHOG (acc. no. P84095; aa 1–178), RHOA (acc. no. P61586; aa 1–181), RHOB (acc. no. P62745; aa 1–181), RHOC (acc. no. P08134; aa 1–181), CDC42 (acc. no. P60953; aa 1–178), TC10 (acc. no. P17081; aa 2–193), RND1 (acc. no. Q92730; aa 1–232), RND2 (acc. no. P52198; aa 26–184), RND3 (acc. no. P61587; aa, 1–244), RIF (acc. no. Q9HBH0; aa 1–195), and mouse RHOD (acc. no. P97348; aa 2–193). pET23b was used to express IQGAP2^C975^ (acc. No. Q13576; aa 780–1575), and IQGAP3^C790^ (acc. No. Q13576; aa 841–1631). Human DOCK DHR2 (acc. no. Q92608; 1211–1624) was cloned in the pOPIN vector as previously described [71].

**Proteins**. All proteins were purified according to established protocols [23]. All proteins, except IQGAP2^C795^, were isolated as glutathione S-transferase (GST) fusion proteins by affinity chromatography on a glutathione Sepharose column in the first step and purified by size-exclusion chromatography after proteolytic cleavage of GST in the second step [23,72]. IQGAP2^C795^ was purified as a His-tagged protein. This protein was isolated from the supernatant via Ni-NTA affinity purification. Nucleotide-free RHO proteins were prepared using alkaline phosphatase (Roche) and phosphodiesterase (Sigma-Aldrich, St. Louis, MO, USA) at 4 °C as previously described [73]. Fluorescent methyl-anthraniloyl (m) was used to generate mGppNHp-bound RHO proteins; GppNHp is a slow-hydrolyzing analog of GTP. The quality and concentrations of the labeled proteins were determined as previously described [73]. RND proteins, which were isolated in the GTP-bound form, could not be loaded with mGppNHp. GTP degradation by alkaline phosphatase, which is normally used to exchange bound nucleotides with other fluorescent nucleotides, led to their precipitation despite the presence of mGppNHp.

**GST pull-down assay.** Confluent HEK293 cells cultured on 10 cm dishes were lysed in lysis buffer containing 50 mM Tris-HCl, pH 7.5; 1%, 150 mM NaCl; and 10 mM MgCl_2_ 1% Igepal Ca-630 supplemented with a protease inhibitor tablet (complete protease inhibitor cocktail, EDTA-free, Merck). 

The cell lysates were poured into prechilled tubes and centrifuged at 13,000 rpm for 5 min at 4 °C. Glutathione agarose beads were washed with ice-cold buffer and incubated with 10 micrograms each of GST-RHOG, GST-CDC42-bound GppNHp, and GST alone for 40 min on a rotator at 4 °C. Then, the samples were centrifuged and washed three times with cold buffer containing 50 mM Tris-HCl, pH 7.5; 1%, 150 mM NaCl; and 10 mM MgCl_2_. The HEK293 cell lysates were added to beads, rotated at 4 °C for 40 min, and later centrifuged and washed three times with cold buffer. The samples were mixed with 1-fold Laemmli buffer, boiled for 10 min at 95 °C and resolved by SDS-PAGE. The separated proteins on 10% SDS-polyacrylamide gels were transferred to a nitrocellulose membrane. The proteins on the membrane were detected by mouse monoclonal anti-IQGAP1 antibody ab56529 (Abcam) and mouse monoclonal anti-GST antibody 2624S (Cell Signaling).

**Analytical size-exclusion chromatography.** CDC42•GppNHp and RAC1•GppNHp were mixed with IQGAP1^C794^ or IQGAP2^C795^ in a buffer containing 30 mM Tris/HCl, pH 7.5; 150 mM NaCl; and 5 mM MgCl_2_. Analyses were performed at a flow rate of 0.5 mL/min and a fraction volume of 0.5 mL on a Superdex 200 10/300 column (GE Healthcare Life Sciences) using an ÄKTA purifier. The *M*_W_s for each eluted peak were calculated based on the calibration curve and the partition coefficient plot (Kav = Ve − V0/Vc − V0) versus the logarithm of the *M*_W_s; Ve, elution volume number; V0, void volume (=8 mL); Vc, geometric column volume (=24 mL). The eluted fractions were collected and resolved by SDS-PAGE, and the gels stained with Coomassie brilliant blue.

**Fluorescence stopped-flow spectrometry.** All kinetic parameters (k_obs_, k_on_, and k_off_) assessed in this study were measured using a previously described kinetic analysis protocol [72]. The kinetic parameters were monitored with a stopped-flow apparatus (Hi-Tech Scientific SF-61 and SX20 MV Applied Photophysics), and the analysis was performed as described [72] using excitation wavelengths of 362 nm (for mant) and 546 nm (for tamra). The emission was detected with a cutoff greater than 408 nm (for mant) and 560 nm (for tamra). The GAP-stimulated GTPase reaction was assessed after 0.2 µM tamraGTP-bound RAC1 was mixed with 10 µM p50^GAP^; tamraGTP is the abbreviation for tetramethylrhodamine-labeled GTP [74]. The GEF-catalyzed nucleotide exchange reaction was assessed after 0.2 µM mGDP-bound RAC1 was mixed with 40 µM GDP and 10 µM TIAM1 or DOCK2. The effector association with the RHO proteins was measured after 0.2 µM mGppNHp-bound RHO proteins were mixed with 2 µM C794 of IQGAP1 or C795 of IQGAP2. Dissociation experiments were performed by displacing the bound effector from the complex upon adding excess unlabeled GppNHp-bound RHO proteins. All measurements were performed in 30 mM Tris-HCl, pH 7.5; 10 mM K_2_HPO_4_/KH_2_PO_4_, pH 7.4; 2 mM MgCl_2_; and 3 mM DTT at 25 °C. The data obtained are averages of at least four independent measurements. Competition experiments were carried out by measuring the association of IQGAP1^C794^ with RAC1•mGppNHp in the presence and absence of excess amounts of RAC-binding proteins: TIAM1 DH-PH, TRIO DH-PH, DOCK2 DHR2, p50^GAP^, PAK1 GBD, plexin B1 RBD, and p67^Phox^ TRP. Previous studies have shown that the association of these proteins with RAC1•mGppNHp, except for IQGAP, does not lead to a change in fluorescence [50,58,65,75], which is a crucial prerequisite for stopped-flow fluorometric competition experiments. The experiment was based on the concept that an increase in fluorescence upon IQGAP association with RAC1•mGppNHp is attenuated by any one of the RAC-binding proteins that compete with IQGAP for the same binding sites. The experimental setup was as follows: syringe 1 contained 4 µM IQGAP1^C794^ and 40 µM of the respective RAC-binding proteins in a premixture, and syringe 2 contained 0.4 µM RAC1•mGppNHp. The two samples were rapidly mixed 1:1 with a dead time of 2 msec and injected into an observation cell at a final volume of 70 µL. The measured rate constants were fitted with a single exponential function using the GraFit program (Erithacus software).

**Structural analysis.** All sequences related to RHO GTPases were retrieved from the UniProt database. Amino acid sequence alignments were performed in the BioEdit program using the ClustalW algorithm [76]. Model structures of RHO GTPases for which no X-ray or NMR structure was available in the PDB were generated with the program MODELLER [77]. The structures generated with MODELLER were RHOG, RIF, RND2, and TCL, and the following PDB entries were used as template structures: 1I4D, 2J1L, 2REX, and 2ATX. Root-mean-square deviation (RMSD) was calculated with the prompt command in the PyMOL program [78]. Structural analysis and electrostatic potential maps were generated using PyMOL molecular viewer, version 1.5.0.4 (Schrödinger, LLC), and the APBS program [78,79,80], respectively. The APBS program is based on a standard procedure in which the electrostatic solvation energies of individual moieties are subtracted from the electrostatic energy of the complex. Protein molecules were fully charged in accordance with the CHARMm force field. The refinement consisted of a short procedure for minimization (200 steps) of the complex energy with the fixed structure of the GTPases. The nonlinear Poisson-Boltzmann equation was used in the calculation of binding energies and in the calculation of the electrostatic potential around the GTPases. To compare the electrostatic potentials of proteins with different total charges, the positive and negative charges of the proteins were scaled separately to ensure that the electrostatic potential of each amino acid was +1 and −1, respectively. A value of electrostatic potential at a certain space point indicates the tendency of an electron (i.e., general negative charge) placed at this point to be repulsed (red) or attracted (blue). Triangulation of the electrostatic isosurfaces for graphic representation was performed using the marching cubes algorithm [81]. The final pictures were generated by the Raster3D package [82]. All APBS calculations were performed with the same parameters: values of electrostatic potential were calculated at the points on the regular 3D grid within the cube with side lengths of 80.0 Å and 60.0 Å for the coarse and fine mesh, respectively, while the number of points in each direction was 128 in both cases. The dielectric constants for the protein and solvent were set to 2.0 and 78.0, respectively.

## Data Availability

All the data are in the manuscript.

## Supplementary Materials

The following are available online at https://www.mdpi.com/article/10.3390/ijms222212596/s1
