# Supplementary material for: Selectivity Determinants of RHO GTPase Binding to IQGAPs
Source: Int J Mol Sci. 2021 Nov 22;22(22):12596. doi: 10.3390/ijms222212596 (PMC8625570; doi:10.3390/ijms222212596)
Supplement: Supplementary file 1 [file ijms-22-12596-s001.zip › ijms-1460011-supplementary.pdf]

Supplementary Data

Selectivity determinants of RHO GTPase binding to IQGAP1

Niloufar Mosaddeghzadeh*, Kazem Nouri*, Oliver H.F. Krumbach, Ehsan Amin, Radovan Dvorsky, M.-Reza Ahmadian

Institute of Biochemistry and Molecular Biology II, Medical Faculty and University Hospital Düsseldorf, Heinrich Heine University Düsseldorf, Düsseldorf, Germany

Table S1. Data summary for the interaction of RHO proteins with IQGAP1 (C794). The dissociation constants (K_d) were calculated from the ratio of the k_{off} values divided by the k_{on} values.

Proteins	k_{on} ($\mu M^{-1}s^{1}$)	k_{off} (s^{-1})	K_d (μM)
**RAC1**	0.70 ±0.0982	0.65 ±0.1212	0.93±0.0943
**RAC2**	2.88 ±0.2001	0.08 ±0.1000	0.027 ±0.0040
**RAC3**	1.53 ±0.0993	0.69 ±0.1387	0.45 ±0.1274
**RHOG**	1.14 ±0.1235	0.56 ±0.0998	0.49 ±0.1635
**CDC42**	1.32 ±0.2099	0.39 ±0.0465	0.29 ±0.2134
**$RAC1^{T25K/N26D}$**	0.13 ±0.0010	2.01 ±0.0470	15.30 ±0.276
**$RAC1^{M45E/N52E}$**	0.17 ±0.0029	1.40 ±0.0395	8.20 ±0.312
**$RAC1^{Q74D}$**	0.12 ±0.005	0.74 ±0.0299	6.10 ±0.241
**$RAC1^{V85D/A88D}$**	0.27 ±0.0033	2.84 ±0.1630	10.50 ±0.263
**$CDC42^{T25K/N26D}$**	0.52 ±0.0109	0.48 ±0.0050	0.92 ±0.110
**$CDC42^{M45E/T52E}$**	0.77 ±0.0142	0.95 ±0.0036	1.23 ±0.092
**$CDC42^{Q74D}$**	0.90 ±0.0125	1.19 ±0.0490	1.32 ±0.075
**$CDC42^{V85D/S88D}$**	0.72 ±0.0066	0.88 ±0.0785	1.2 ±0.090
**$RHOA^{K27T/D28N/E47M/E54N/D76Q}$**	1.20 ±0.0645	2.10 ±0.2170	1.75 ±0.180

The kinetic data are shown in Figures S2 and S5, and illustrated as bar charts in Figure 1B.

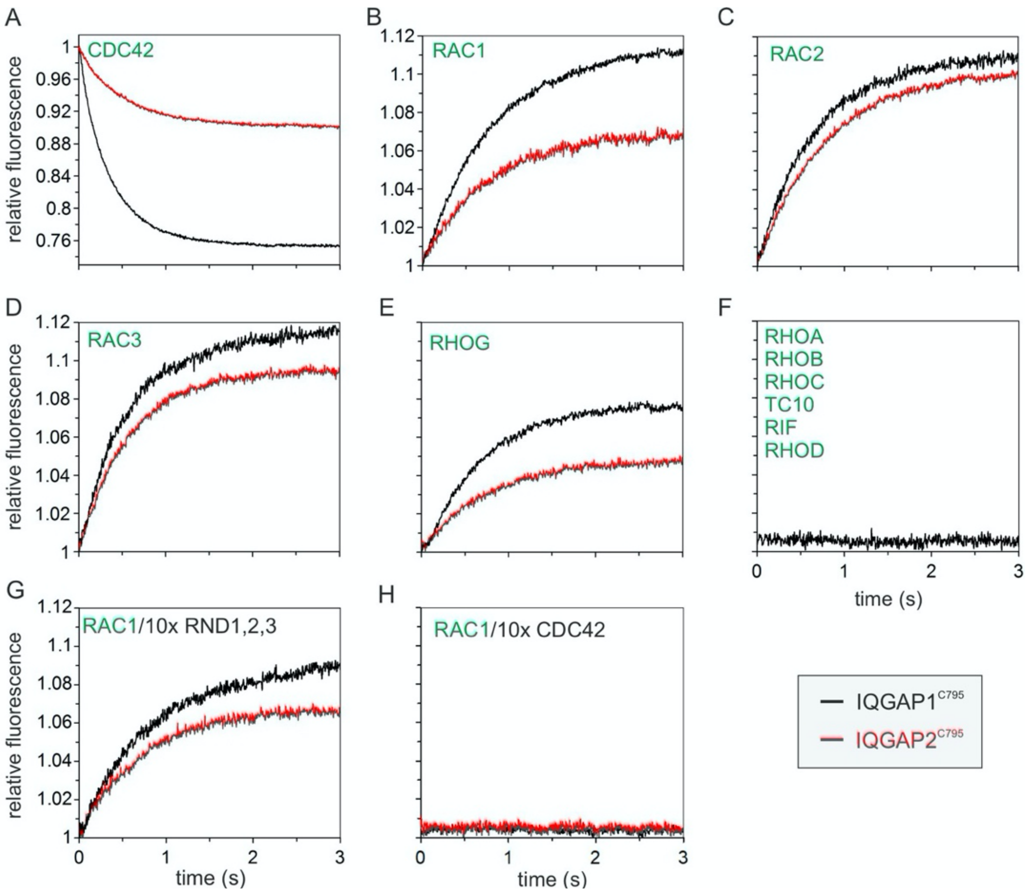

Figure S1. Kinetics of IQGAP1/2 association with different RHO GTPases. (**A-E**) CDC42 and RAC-like proteins are IQGAP1/2 binders. Association of 2 µM IQGAP1^{C794} (black) and 2 µM IQGAP2^{C795} (red) with mGppNHp-bound RHO family proteins (0.2 µM). As previously described (see Ref. 23), IQGAP1 binding to CDC42•mGppNHp resulted in a fluorescence decay whereas IQGAP1 association with mGppNHp-bound RAC-like proteins led to an increase in fluorescence. This supports the notion that CDC42 and RAC1, despite their high sequence identity (71%), obviously differ in regard to their binding modes with IQGAPs (see ref. 40). Moreover, IQGAP1 and IQGAP2 association particularly with CDC42 resulted in similar rates (see Fig. 1B), but different amplitudes, which may indicate a deviation in the binding properties of the two IQGAP paralogs. **(F)** The IQGAP1/2 non-binders. The graph shows the results of mixing 0.2 µM RHOA•mGppNHp with IQGAP1^{C794} as a representative for a lack of interaction (no change in fluorescence) for RHOB, RHOC, TC10, RHOD and RIF. The same results were obtained for IQGAP2^{C795}. **(G)** RND proteins group also belong to the IQGAP1/2 non-binders. As GTP-binding proteins, RND1-3 could not be loaded with mGppNHp. A competition assay was performed to measure an association with the IQGAP paralogs. Association of 2 µM IQGAP1^{C794} (black) and 2 µM IQGAP2^{C795} (red) with 0.2 µM RAC1•mGppNHp was measured in the presence of 10-fold excess amount of GTP-bound RND1, RND2 or RND3 (10 µM, respectively). The association of IQGAPs with RAC1•mGppNHp resulted in an increase of fluorescence, which clearly indicate that the RND proteins do not bind to IQGAP1 and IQGAP2. Evaluated observed rate constants (k_{obs}) of these data are illustrated as bar charts in Figure 1B. **(H)** Association of 2 µM IQGAP1^{C794} (black) and 2 µM IQGAP2^{C795} (red) with 0.2 µM RAC1•mGppNHp was measured in the presence of 10-fold excess amount of GppNHp-bound CDC42 (10 µM, respectively). Competitive binding of the IQGAPs to CDC42•GppNHp completely abolished their association with RAC1•mGppNHp. Evaluated observed rate constants (k_{obs}) of all data are illustrated as bar charts in Figure 1B, where the green barcharts belong to IQGAP1^{C794} measurements and blue to IQGAP2^{C795}.

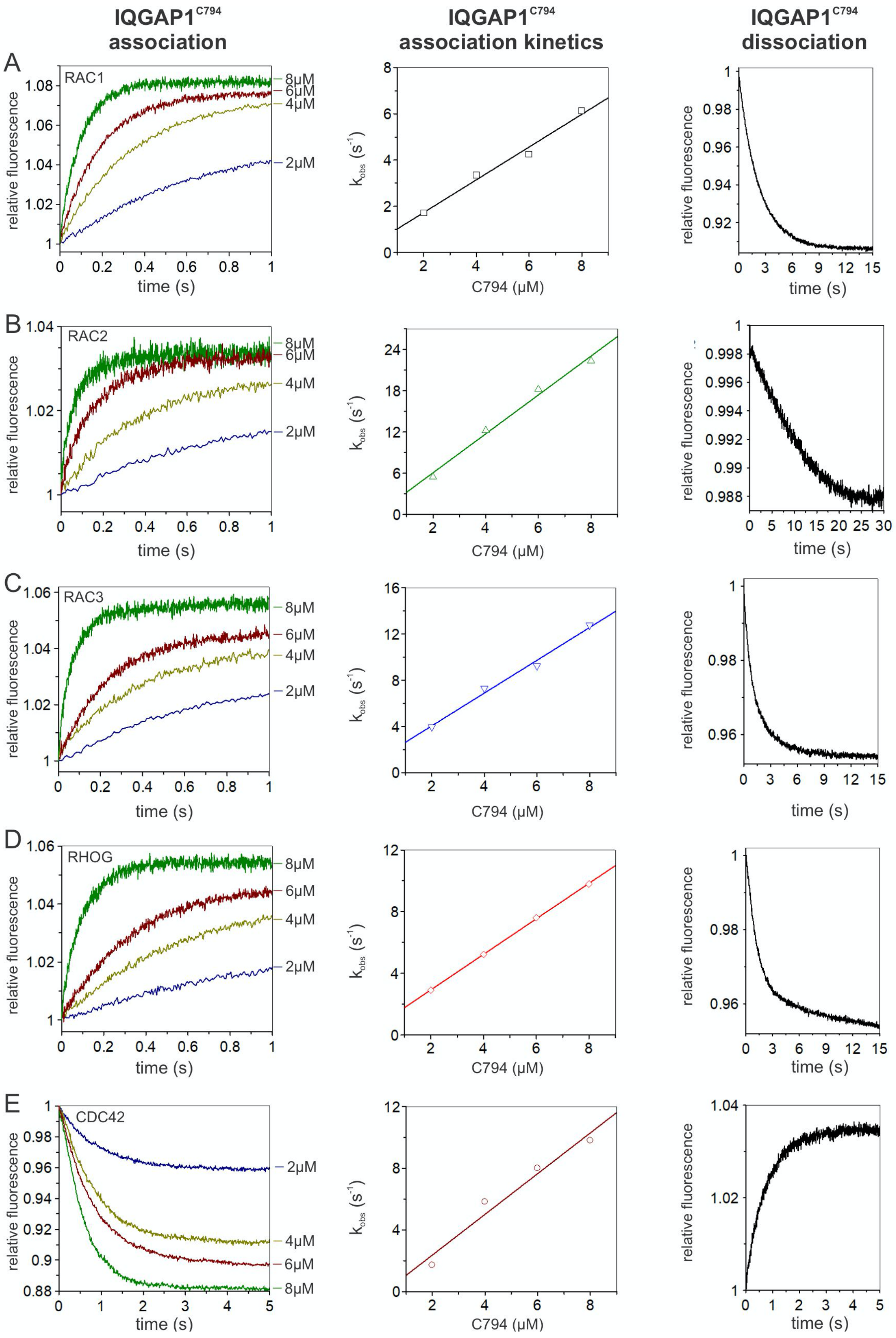

Figure S2. Kinetic measurements of IQGAP1^{C974} interaction with RHO family proteins. Left panels: Association of mGppNHp-bound CDC42/RAC-like proteins (0.2 μM) with increasing IQGAP1^{C794} concentrations (2-8 μM). Middle panels: Evaluated association rate constant (k_{on}) from the plot of the k_{obs} values, obtained from the exponential fits to the association data (left panels) against the corresponding IQGAP1^{C794} concentrations. Right

panels: Dissociation of IQGAP1^{C794} (2 µM) from mGppNHp-bound CDC42/RAC-like proteins (0.2 µM) in the presence of excess amounts of unlabeled GppNHp-bound CDC42/RAC-like proteins (10 µM). The results are compiled in Table S1 and illustrated as bar charts in Figure 1C.

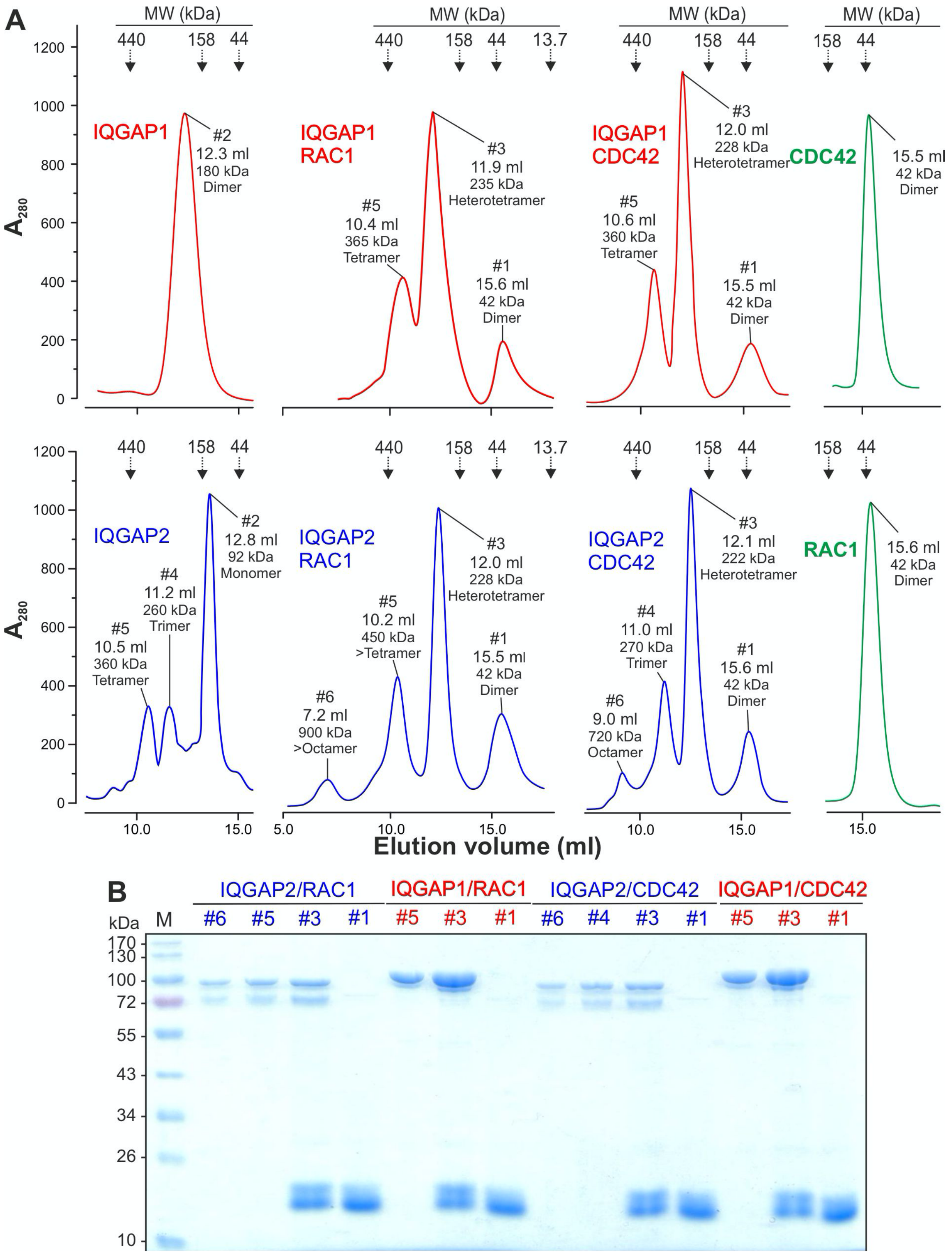

Figure S3. IQGAP form heterotetrameric complexes with RAC1 and CDC42. (A) Analytical size exclusion chromatography of IQGAP1^{C794} (red) and IQGAP2^{C795} (blue) alone and in a mixture with RAC1•GppNHp and CDC42•GppNHp was performed on Superdex 200 10/300 column (GE Healthcare Life Sciences), using an ÄKTA purifier (flow rate of 0.5 ml/min, fraction volume of 0.5 ml) and a buffer, containing 30 mM Tris/HCl, pH 7.5, 150 mM NaCl, 5 mM $MgCl_2$. The column was calibrated using a calibration kit (GE Healthcare Life Sciences), containing Ferritin (440 kDa), Aldolase (158 kDa), Ovalbumin (44 kDa), and Ribonuclease (13.7 kDa). The elution profiles revealed six peaks: (#1) at 15.5 to 15.6 ml for dimeric RAC1 and CDC42 with a molecular weight (MW) of 42 kDa, as also shown in the right panel; (#2) at 12.8 to 12.3 ml for monomeric IQGAP2 and dimeric IQGAP1 with MWs of 92 and 180 kDa, respectively;

(#3) at 12.1 to 11.9 ml for a heterotetrameric complex of the IQGAP1/2 complexes with RAC1/CDC42 with MWs of 222-235 kDa, respectively (see B); #4 at 11.0 to 11.2 ml for trimeric IQGAP2 with MWs of 260-270 kDa; #5 at 10.2 to 10.6 ml for tetrameric IQGAPs with MWs of 360-450 kDa; #6 at 7.2 to 9.0 ml for octameric IQGAPs with MWs of 720-900 kDa. The theorical MWs for RAC1 and CDC42 of 21.4 and 21.2 kDa, respectively, were taken from Uniprot database. A MWs of 90 kDa was calculated for IQGAP1^{C794} and IQGAP2^{C795} using the Expasy tool. Indicated MWs for each peak were calculated based on calibration curve and the partition coefficient plot (Kav = Ve-V0/Vc-V0) *versus* the logarithm of MWs; Ve: elution volume number; V0 void volume (= 8 ml); Vc: geometric column volume (= 24 ml). **(B)** A Coomassie brilliant blue stained SDS-PAGE showed that only peaks #3 at an elution volume of 12.1/11.9 ml contain the IQGAP1/2 complexes with RAC1/CDC42.

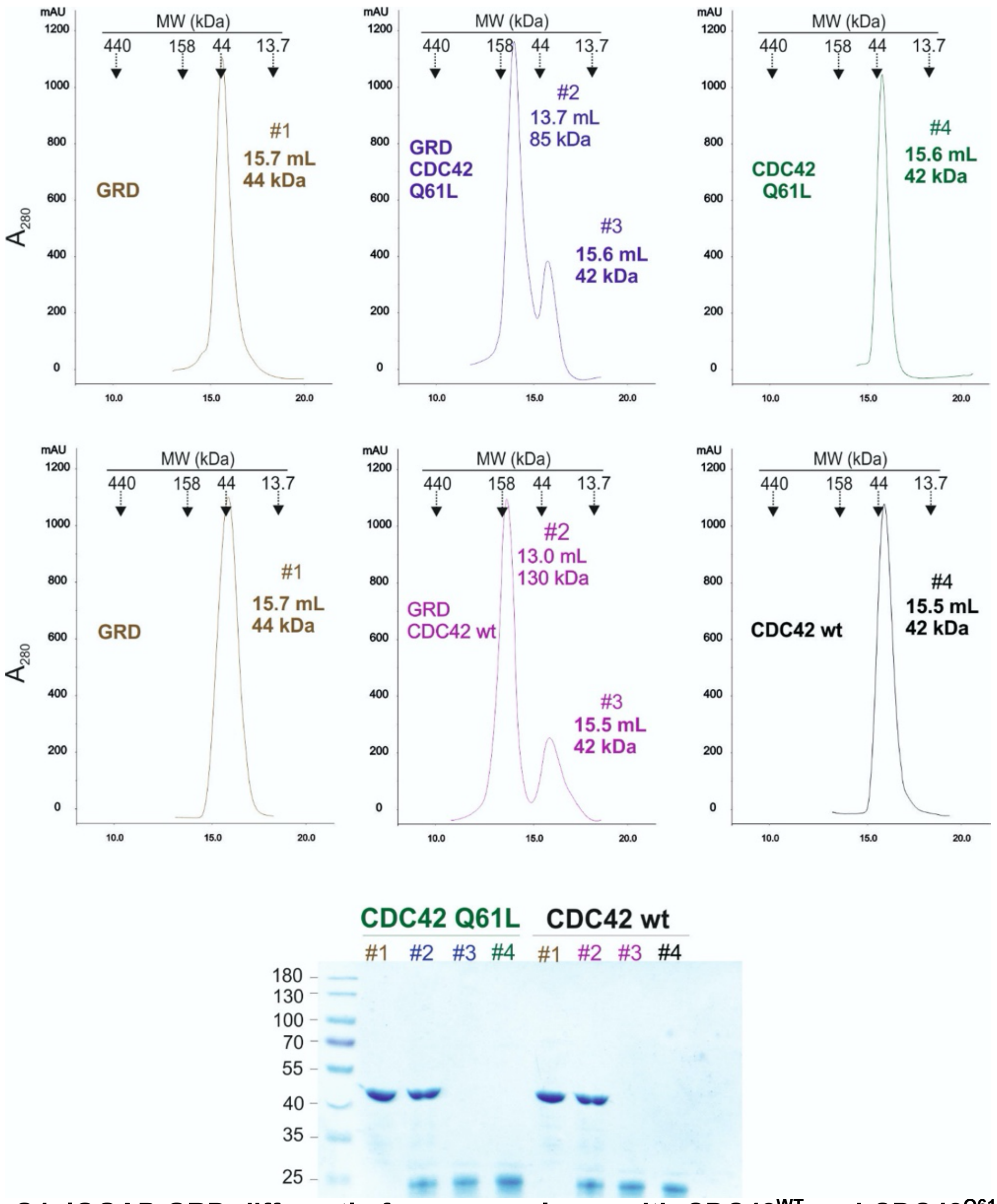

Figure S4. IQGAP GRD differently forms complexes with CDC42^{WT} and CDC42^{Q61L}. The analytical size exclusion chromatography (Superdex 200, 10/300) analysis was performed by mixing CDC42^{Q61L}•GppNHp (upper panel) or CDC42^{WT}•GppNHp (lower panel) with IQGAP1 GRD using an ÄKTA purifier (flow rate of 0.5 ml/min, fraction volume of 0.5 ml) and a buffer, containing 30 mM Tris/HCl, pH 7.5, 150 mM NaCl, 5 mM $MgCl_2$. Under the same conditions as described in Figure S3. In the case of CDC42^{Q61L}, the elution profile represented two peaks (upper panel) for the GRD and CDC42^{Q61L} complex (#2) at 13.7 ml corresponding to heterotrimeric complex of GRD and CDC42^{Q61L} with a molecular weight of 85 kDa and peak #3 at 15.6 ml for dimeric CDC42^{Q61L} around 42 kDa. However, the elution profile of GRD and CDC42^{WT} complex (middle panel) showed complex elution as a heterotetramer (#2) at 13.0 mL with a molecular weight of 130 kDa, and a dimeric CDC42^{WT} (#3) at 15.5 mL. The theoretical MWs of CDC42 (21.2 kDa) and GRD (43 kDa) were calculated using the Expasy tool. The presented MWs for each peak were calculated based on the calibration curve and partition coefficient plot (Kav = Ve-V0/Vc-V0) versus the logarithm of MWs; Ve: elution volume number; V0: void volume (8 mL); Vc: geometric column volume (24 mL). A Coomassie brilliant blue staining of the corresponding elution volumes indicated that only peaks #2 at 13.7/13.0 mL contain GRD complexes with CDC42 WT and Q61L, respectively (lower panel).

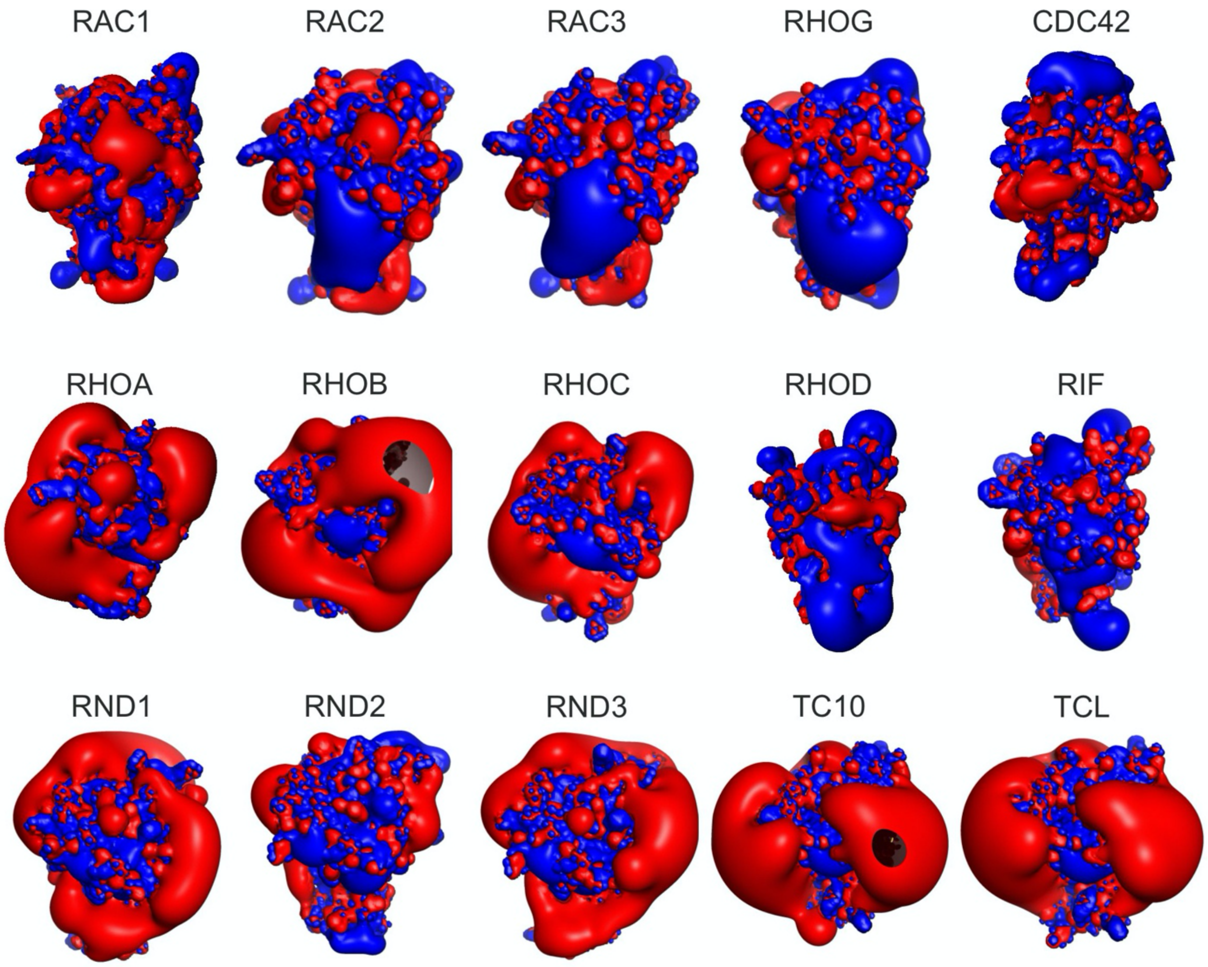

Figure S5. RHO GTPases significantly differ in their electrostatic potentials. Electrostatic potential maps of 15 RHO GTPases, including RAC1 (PDB code: 1MH1), RAC2 (PDB code: 2W2V), RAC3 (PDB code: 2IC5), CDC42 (PDB code: 2QRZ), RHOA (PDB code: 1A2B), RHOB (PDB code: 2FV8), RHOC (PDB code: 2GCO), RND1 (PDB code: 2CLS), RND3 (PDB code: 1M7B), RHOD (PDB code: 2J1L), and TC10 (PDB code: 2ATX) are represented by their isosurfaces at −0.1 kb T/e c (red) or +0.1 kb T/e c (blue), respectively. The structures of RIF, RHOG, RND2, and TCL were modelled using SWISS-MODEL. PyMOL molecular viewer was used for the analysis and illustrations (see experimental procedures). The orientation of the molecules is the same as in Figures 2C and 3D. As all modelled structures are highly homologous to their templates, RMSD of resulted models to corresponding template structures are very low (under 0.30 Å), particularly 0.22 Å for RIF, 0.21 Å for RHOG, 0.18 Å for RND2 and 0.19 Å for TCL.

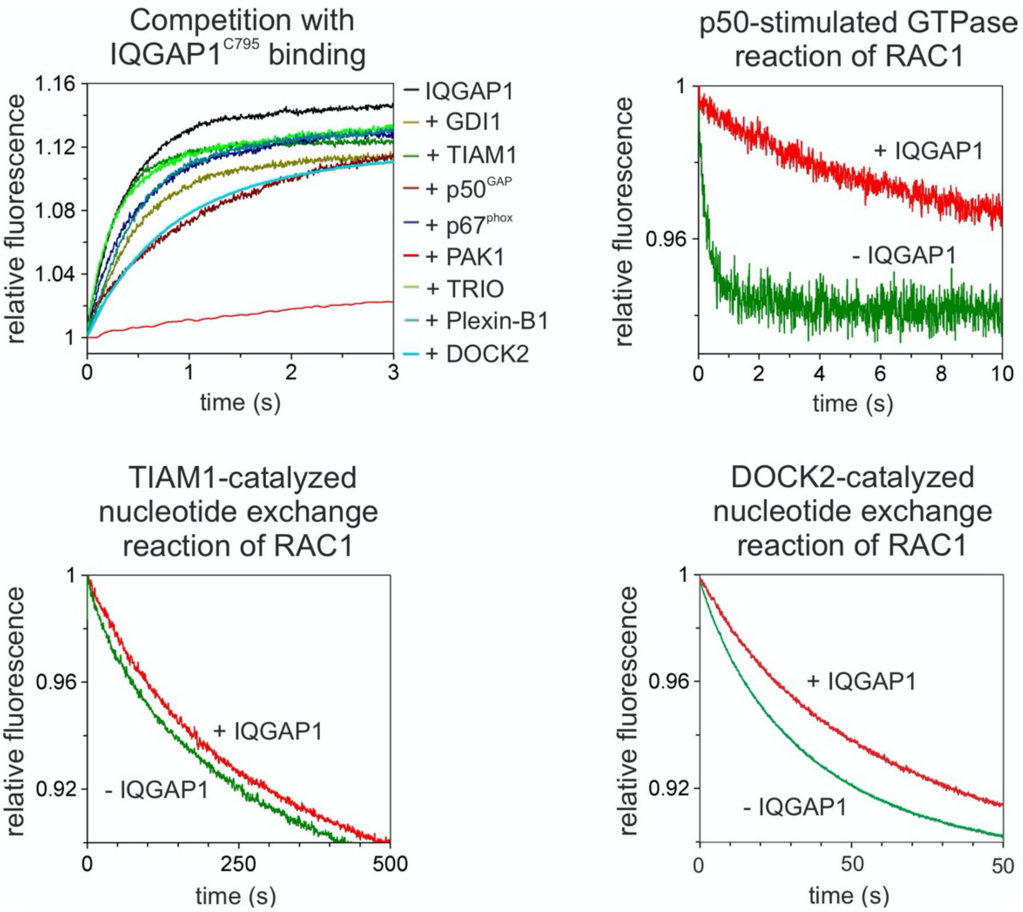

Figure S6. DOCK2, p50GAP and PAK1 compete with IQGAP1^{C794} for binding to RAC1. (**A**) Association of IQGAP1^{C794} (2 µM) with mGppNHp-bound RAC1 (0.2 µM) was measured in the presence of excess amount of the RAC1- and CDC42-interacting partners (20 µM). (**B**) GAP-stimulated GTP hydrolysis of RAC1 was measured using 0.2 µM RAC1•tGTP (tGTP stands for tetramethylrhodamine-labelled GTP) and 10 µM p50^{GAP} in presence and absence of 100 µM IQGAP1^{C794}. (**C**) The GEF-catalyzed nucleotide exchange reaction was measured using 0.2 µM RAC1•mGDP (mGDP stands for N-methylanthraniloyl-labelled GDP) and 200 µM TIAM1 or DOCK2 in the presence and/or absence of 100 µM IQGAP1^{C794}. Evaluated data are shown as bar charts in Figure 3A-C.

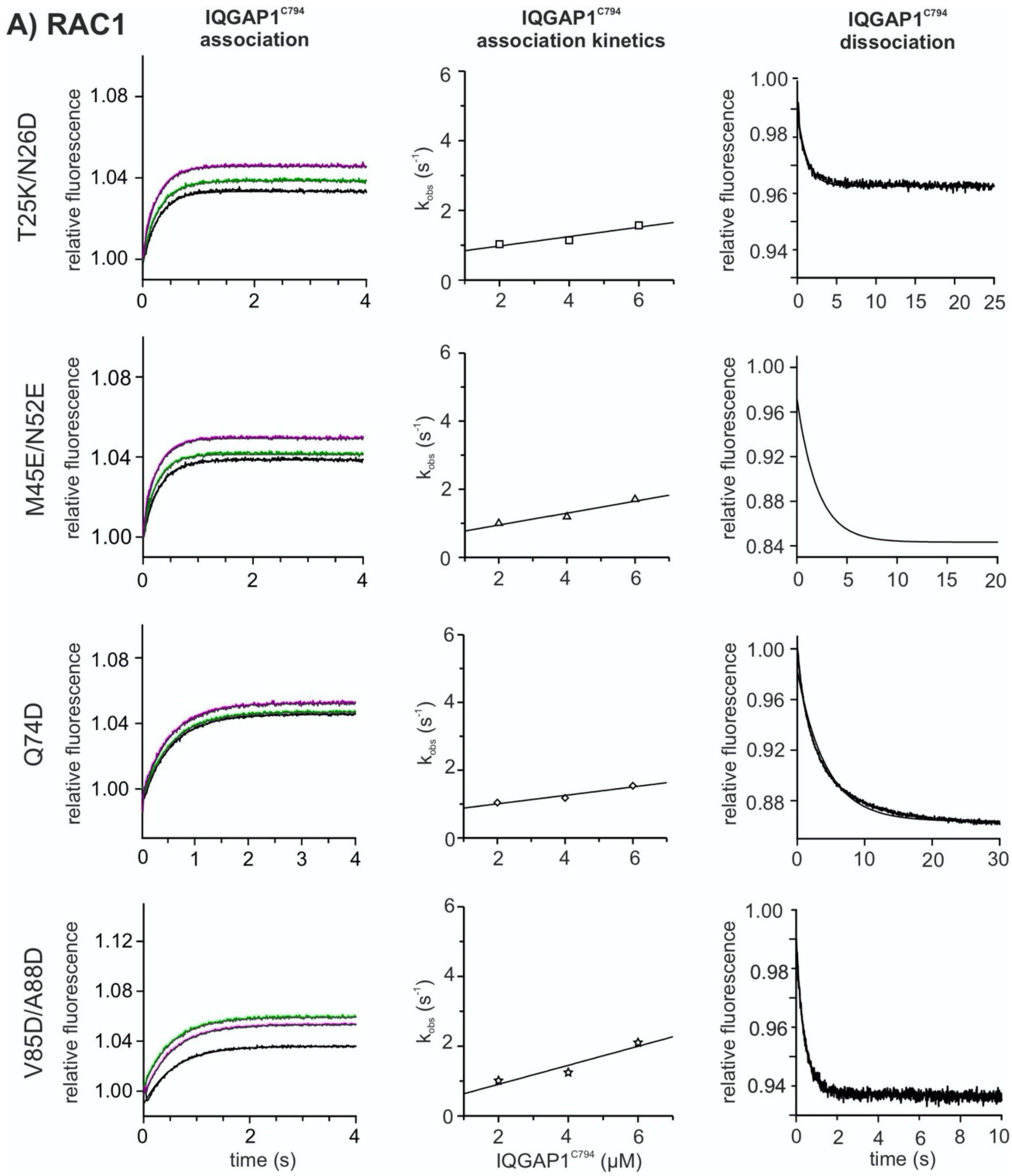

A) RAC1
IQGAP1C794 association
IQGAP1C794 association kinetics
IQGAP1C794 dissociation
T25K/N26D
M45E/N52E
Q74D
V85D/A88D
relative fluorescence
kobs (s-1)
time (s)
IQGAP1C794 (µM)

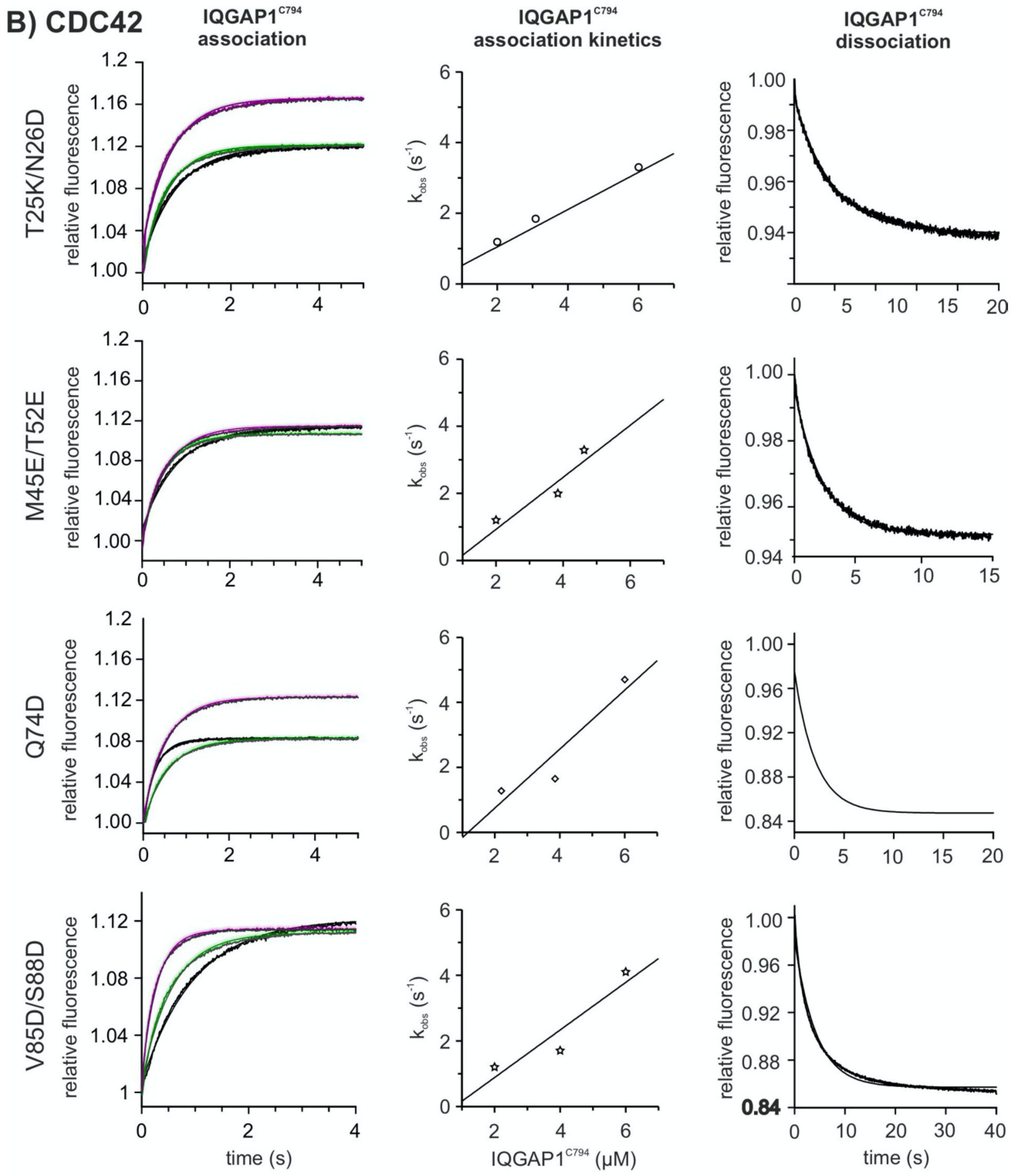
B) CDC42
IQGAP1C794 association
IQGAP1C794 association kinetics
IQGAP1C794 dissociation
T25K/N26D
M45E/T52E
Q74D
V85D/S88D
relative fluorescence
kobs (s-1)
time (s)
IQGAP1C794 (µM)

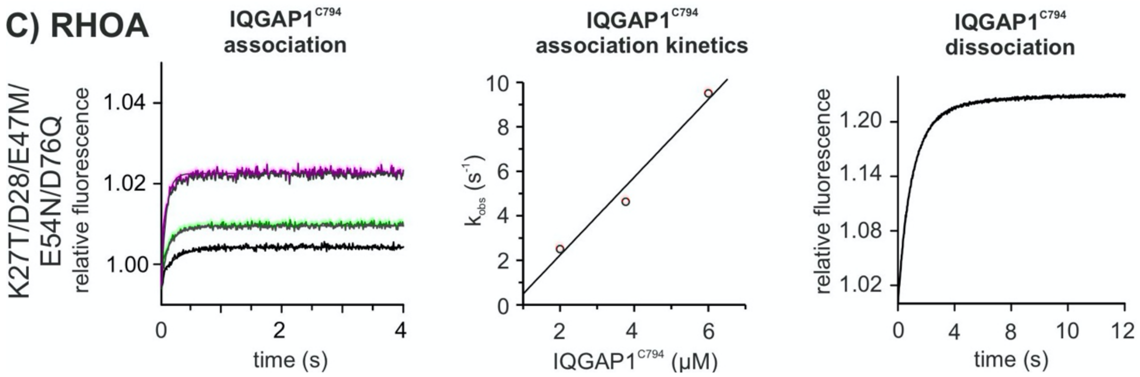

Figure S7. Kinetic measurements of IQGAP1^{C974} interactions with the variants of RAC1 (A), CDC42 (B), and RHOA (C). Left panels: Association of mGppNHp-bound proteins (0.2 µM) with increasing IQGAP1^{C795} concentrations (2, 4 and 6 µM, respectively, in black, green and magenta). Middle panels: Evaluated association rate constant (k_{on}) from the plot of the k_{obs} values, obtained from the exponential fits to the association data (left panels) against the corresponding IQGAP1^{C794} concentrations. Right panels: Dissociation of IQGAP1^{C794} (2 µM) from 0.2 µM mGppNHp-bound RAC1, CDC42 and RHOA variants in the presence of excess amounts of 10 µM unlabeled GppNHp-bound wildtype RAC1, CDC42 and RHOA. The results are compiled in Table S1 and illustrated as bar charts in Figure 4.
